# Case Report: Full-Endoscopic Surgery for Bullet Wounds of the Spine: A Report of Three Cases

**DOI:** 10.3389/fsurg.2022.873365

**Published:** 2022-03-25

**Authors:** Maxim N. Kravtsov, Vadim A. Manukovsky, Gennadiy G. Bulyshchenko, Saidmirze D. Mirzametov, Vadim A. Byvaltsev

**Affiliations:** ^1^Department of Neurosurgery, S.M. Kirov Military Medical Academy, St. Petersburg, Russia; ^2^Saint-Petersburg I.I. Dzhanelidze Research Institute of Emergency Medicine, St. Petersburg, Russia; ^3^Department of Neurosurgery, North-Western State University n.a. I.I. Mechnikov, St. Petersburg, Russia; ^4^Department of Neurosurgery, Irkutsk State Medical University, Irkutsk, Russia; ^5^Department of Neurosurgery, Railway Clinical Hospital, Irkutsk, Russia

**Keywords:** gunshot wound, injury, lumbar spine, thoracic spine, full-endoscopic surgery

## Abstract

**Objectives:**

To determine the feasibility and evaluate effectiveness of full-endoscopic surgery in gunshot wound of the spine.

**Methods:**

Three clinical cases of lumbar and thoracic spine bullet wounds made by firearms and traumatic weapons are described. Percutaneous endoscopic surgery was performed to extract bullet from the spinal canal. The results are compared to the data from literature.

**Results:**

Percutaneous endoscopic approach to spinal canal with a possibility to extract a bullet, decompression of nerve roots, defect closure of the dura mater is demonstrated.

**Conclusion:**

Good clinical outcomes allows to recommend percutaneous endoscopic surgery to manage similar lumbar and thoracic spine bullet wounds at the tertiary care level.

## Introduction

The potential of full-endoscopic surgery has greatly improved thanks to advanced video transmission quality, upgrades of endoscopes and related instruments and the development of new surgical techniques and approaches. It all resulted in expanding indications for this type of treatment (1, 27). However, degenerative-dystrophic diseases of the spine still remain the main pathology where percutaneous endoscopic interventions are largely used (2). Also, a beneficial effect of the described method was noticed in revision surgery after metal osteosynthesis (3), in non-specific spondylodiscitis (4) and spinal tumors (5, 6).

This paper assesses preliminary results of percutaneous video endoscopy for gunshot bullet wounds in the lumbar spine; presents capabilities of surgical technique for extraction of a foreign body from the spinal canal and intervertebral disc. Treatment of traumatic spinal injuries logically originates from percutaneous video endoscopic spinal surgery.

## Clinical Case No. 1

The wounded person, a 24-year-old man, was admitted to the neurosurgery clinic the next day after he had received a gunshot blind wound in the lumbar region. The patient complained of weakness in the feet, numbness on the back surface of both legs and perineum, impaired sensation of bladder filling. These effects occurred immediately after the injury. During the day, weakness in the right foot increased. Upon admission, the examining physician saw an inlet of the gunshot wound, 10 × 5 mm in size, located in the lumbar region, 6 cm left to the spinous processes line. In the history, there was no intense bleeding or fluid leakage from the wound inlet. The general condition of the patient remained stable, urination passed through the urinary catheter, with normal urine output and normal color of the urine. Neurological status: low flaccid distal paraparesis up to 3 points, bilateral absence of Achilles reflexes, anesthesia in S1-S5 dermatomes, urinary retention.

**Figure 1:**
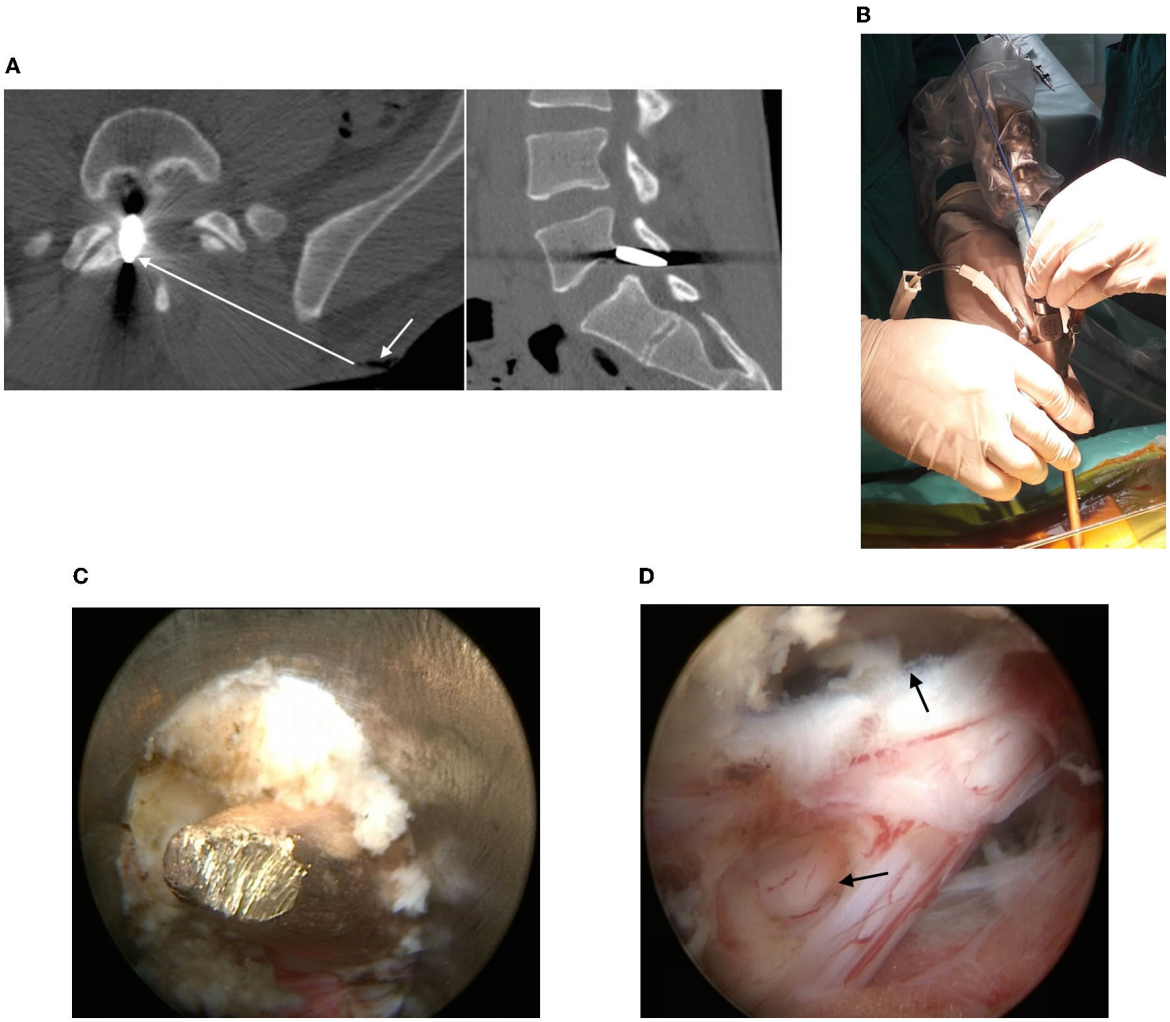
**(A)** CT scans of the lumbar spine: metallic foreign body (bullet) in the right half of the spinal canal (the arrow shows the inlet of the gunshot wound, the line shows an approximate trajectory of the bullet through the soft tissues); **(B)** View of the working port and endoscope; **(C)** Bullet mobilization in a yellow ligament defect; **(D)** Defects in the dura mater around the nerve root cuff and dural sac (see arrows).

Computed tomography confirmed a left-sided wound inlet and channel with an oblique trajectory that ended blindly in the spinal canal close to the right L5-S1 intervertebral joint, where CT picture showed a foreign metal body—a bullet. There were no bone injuries in the spine or impaired large vessels and internal organs in the abdominal cavity, retroperitoneal space and small pelvis. Apart from the bullet, there were no other signs of neural structures compression (Figure 1A).

Results of the examination proved that neurological disorders were likely to be caused by direct trauma to the *cauda equina* and persistent roots compression by the injuring body. The purposes of surgical intervention were extracting the bullet and revising structures of the spinal canal with the help of percutaneous video endoscopy. Should it appear impossible to achieve those purposes, it was planned to convert to open access.

### Surgery

After general anesthesia induction the patient was prone positioned; under fluoroscopic guidance in AP view, a puncture access with an 18G needle was made to the arch of the L5 vertebra 1 cm right to the spinous processes line. The access trajectory did not coincide with the gunshot wound projection. A wire guide was inserted, and a linear cut up to 1 cm long was made. Along the guide, with the help of tubular expanders, a working tube with an outer diameter of 8 mm was inserted through the cut. A SpineTip endoscope (Karl Storz, Germany) was inserted into the working tube (Figure 1B).

Further manipulations were controlled by video endoscopy backed by continuous irrigation with saline sodium chloride solution through a special endoscopic channel. The lower edge of the L5 vertebra arch was skeletonized and partially resected with a diamond burr in order to increase the interlaminar space. The revision showed a distal end of the bullet within the defect of the yellow ligament. With the help of video endoscopy, the working tube axis was aligned with the bullet axis. After partial flavotomy, the bullet was captured with forceps and removed through the lumen of the working port together with the endoscope. Revision of the epidural space showed defects of the dura mater, endoscopic analysis proved anatomical integrity of the roots. The epidural space was revised above and below the injury area. Defects in the dura mater were covered with Fibrin-collagen patch TachoComb^®^ introduced through the working port. Each stage of the surgery is shown in Figures 1C,D and Supplementary Video 1.

A temporary stop of irrigation helped to detect endoscopic signs of unstable cerebrospinal fluid stasis. The endoscope and working port were removed. The skin wound was sutured. The surgery lasted 40 min. Blood loss was about 10 ml. There were no perioperative complications. Postoperative and gunshot wounds healed within 10 days supported by antibiotic therapy (Ceftriaxone 2.0 g per day). There was no cerebrospinal fluid leakage.

Size of the bullet: caliber 5.45 mm, length 23 mm. Postoperative computed tomography and magnetic resonance imaging showed the absence of the foreign body in the spinal canal and restoration of the subarachnoid space patency (Supplementary Video 1).

Within 3 months, the patient regained strength in his left foot. Paresis of the right foot flexors remained at the grade 4. Disorders of urination and defecation completely regressed, cutaneous sensation and sexual function were restored. Back pain was not a concern.

## Clinical Case No. 2

A 39-year-old man referred to the neurosurgery clinic with back pain associated with recurrent retroperitoneal phlegmon. He reported that 13 years ago he had received a gunshot—a penetrating blind wound of the abdomen with a damage to the liver, gallbladder, duodenum, colon, L1-L2 vertebral bodies. The bullet had landed in the L1-L2 intervertebral disc. There had been no neurological disorders.

**Figure 2:**
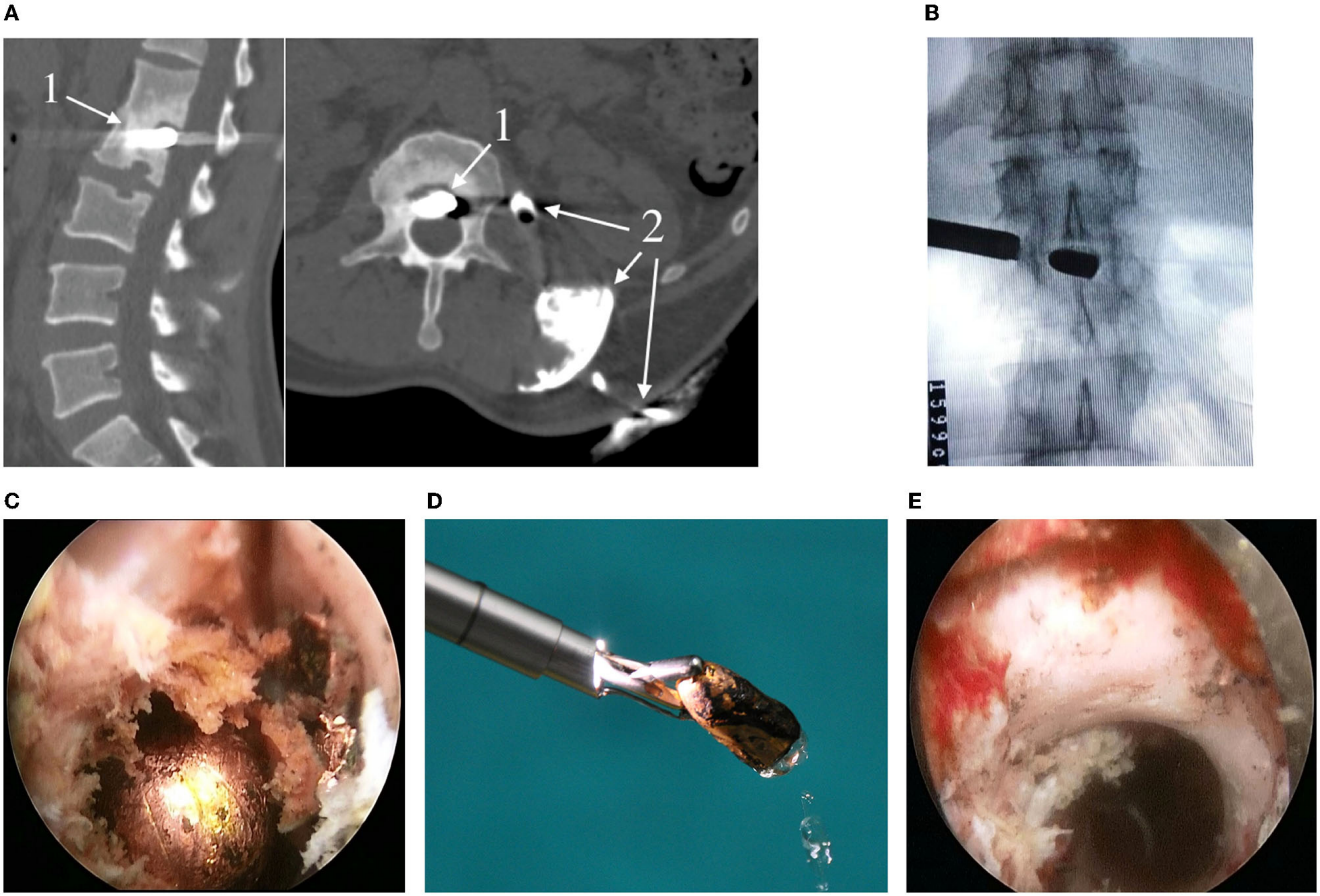
**(A)** Preoperative sagittal CT (left) and CT fistulography (right) of the lumbar spine: 1—bullet; 2—contrast agent; **(B)** Radiography of the working port and bullet; **(C)** Endoscopic stage of the surgery and view of the bullet (described in the text); **(D)** Capturing and extracting the bullet with forceps (caliber 7.62 mm, length 15 mm); **(E)** Endoscopic view of the intervertebral foramen after extracting the bullet.

In order to eliminate consequences of the injury the patient received a staged surgical treatment on the abdominal organs; however, the surgeons had refrained from removing the bullet. The patient had fully recovered. During the last year, the patient suffered from recurrent retroperitoneal phlegmon accompanied by febrile fever and intense lumbar pain; his treatment involved four openings and drainages of purulent foci through retroperitoneal access. CT fistulography showed a thin fistulous tract between the retroperitoneal abscess cavity and the foreign body at the level of L1-L2 vertebrae (Figure 2A).

It was suggested that a probable cause of recurrent infectious process was a bullet, so it was decided to remove a foreign body from the spine through percutaneous transforaminal endoscopic access.

### Surgery

After general anesthesia induction the patient was prone positioned; under fluoroscopy control in AP and lateral views, an 18G needle and a wire were inserted in the lower part of the left intervertebral foramen L1-L2. At the puncture site, a 1.0 cm long transverse incision of the skin and soft tissues was made. A cone-shaped guide and a working tube were inserted along the wire. The guide and wire are removed (Figure 2B). A SpineTip endoscope (Karl Storz, Germany) was inserted into the working tube. Further surgery was controlled by video endoscopy, which visualized bone markers of the intervertebral foramen and anterior epidural space. Fragments of the intervertebral disc back were removed. The bullet was detected. The bullet shell was destroyed, and difficult to separate from the surrounding tissues, which have a dirty gray color and numerous metal inclusions (Figure 2C). In order to form a channel for mobilization and extraction of the bullet, the lower edge of the L1 vertebra body was partially resected with a high-speed burr. The bullet was mobilized with hooks and scoops, fixed with forceps, and removed together with the working tube (Figure 2D). Under fluoroscopic control, the working tube and endoscope were reinserted transforaminally into the L1-L2 intervertebral disc, where there were many bullet shell fragments, removed with forceps and cutters (Figure 2E). Some fragments of the shell with surrounding soft tissues were taken for bacteria culture tests. Hemostasis was controlled by bipolar coagulation. After the last revision of the surgical wound, the working tube and the endoscope were removed. The skin wound was sutured with an interrupted suture. Surgery blood loss was <20 ml. During the surgery, the patient received antibiotic therapy with Vancomycin 1.0 g intravenously. The surgery lasted for 50 min (Supplementary Video 2).

The patient was mobilized the next day after the surgery. For 2 days he had an increased body temperature to 37.8°C, then the temperature got back to normal. The patient received antibacterial therapy (Cefazolin 2.0 g) for 7 days. The bacteria culture test of the sample taken during the surgery revealed *Escherichia coli*, sensitive to most antibiotics. On the 7th day after the surgery, the patient was discharged from the hospital. Upon discharge, he had no complaints; the neurological status was at the preoperative level.

## Clinical Case No. 3

**Figure 3:**
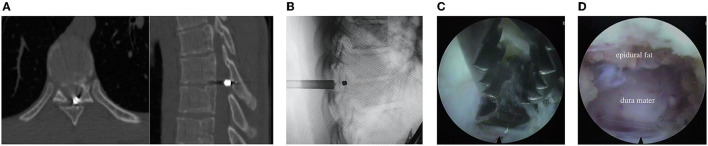
**(A)** CT scans of the thoracic spine (explained in the text); **(B)** Radiography of the working port and bullet; **(C,D)** Endoscopic stage of the surgery (described in the text).

A 19-year-old man was admitted to the neurosurgery clinic with a bullet wound in the back from an air gun. Upon admission he complained of thoracic spine pain. A physical examination of the thoracic spine showed an inlet with a size 0.3 × 0.5 mm. There were no signs of cerebrospinal fluid leakage and bleeding, no neurological disorders. X-ray of the chest in the Th8-Th9 vertebrae showed a foreign body—a bullet. CT scan of the thoracic spine also showed a metal-density foreign body in the spinal canal under the lamina of the Th8 vertebral arch (Figure 3A).

It was decided to make a full-endoscopic intervention to extract the foreign body from the spinal canal at the level of Th8-Th9 vertebrae.

### Surgery

After general anesthesia induction the patient was prone positioned; under fluoroscopy control in AP and lateral views at the level of the Th8 vertebra arch, 2 cm outward and left to the midline, an 18G needle and a wire were sequentially inserted slightly above the bullet wound inlet. The needle was removed. Through a skin incision 1.0 cm long, a cone-shaped guide was inserted along the wire; a working tube was inserted along the cone-shaped guide, and an endoscope (Joimax, Germany) was inserted into the working tube (Figure 3B). With the help of a diamond burr, the arch of the Th8 vertebra was resected in a limited area. Within the yellow ligament there was a rounded defect, in its lumen a bullet was seen. After additional flavotomy, the bullet was removed from the spinal canal with the help of forceps (Figure 3C). Through the defect of the yellow ligament, the endoscope was introduced into the epidural space. The dura mater of the spinal cord showed no signs of impairment (Figure 3D). The dural sac had a distinct pulsation. Hemostasis was supported by bipolar coagulation. After a last revision of the surgical wound, the working tube and the endoscope was removed. The skin wound was sutured with an interrupted suture. The blood loss was <10 ml. The surgery lasted 40 min.

The patient stayed in the hospital for 3 days. At the time of discharge, the patient had no complaints. Neurological status was at the preoperative level.

## Discussion

Gunshot wounds of the spine and spinal cord in peacetime and wartime make 10–21% of all spinal injuries (7–9). In 2014 in the United States, 16.8% (33,594 people) of deaths from injuries were associated with damaging effects of firearms (10). Males aged 15–34 years are more likely to be affected by this type of injury (various authors, 78–91%), 10–24.5% are lumbar spine injuries, of which penetrating injuries make about 14% (9, 11–14). Gunshot wounds of the spine are often accompanied with injuries to the neck, chest, abdominal cavity and retroperitoneal space. A key factor to make prognosis in the acute and early periods of combined injuries is emergency surgery on the damaged vessels and organs (13).

So far, there is no generally approved medical care algorithm for such patients. Such factors as indications amount, reason and time of surgical intervention remain relevant (9, 15–17). A standard procedure in diagnosing spine gunshot wounds is computed tomography, which allows assessing position of the bullet and degree of bone damage (15).

Surgical treatment of spine gunshot wounds is needed in the following cases: increased neurological deficit, neural structures compressed by a bone fragment, intervertebral disc or foreign body, cerebrospinal fluid leakage, gunshot penetrating blind injury to the spinal cord cone and cauda equina, spinal instability, infectious complications and pain syndromes in the late injury period (18, 19). The purpose of the surgery in penetrating blind wounds is to remove a foreign body, decompress neurovascular lesions of the spinal canal, and restore integrity of the dura mater and patency of the subarachnoid space (15).

Despite the obvious indications listed above, the effectiveness of surgical treatment of gunshot wounds to the spinal cord remains low. Treatment outcomes for those with gunshot wounds to the cervical and thoracic spine, in the absence of positive dynamics in neurological status, did not differ between conservative and surgical groups (16).

The necessity of removal of a wounding body in uncomplicated non-penetrating gunshot wounds of the spine, especially in the late period, is still open for discussion. Experts differ on the toxic effects of lead when a wounding body remains for a long time (10, 20); however, there is no doubt that a foreign body must be removed in cases of purulent-inflammatory complications (21).

Surgeons should carefully select an access to the spine for bullet removal, and be guided by the position of the bullet against parts of the spinal canal and neurovascular structures. The most common and universal method for accessing a bullet in the spinal canal is laminectomy (9). Lateral and anterior approaches are typical for the removal of foreign bodies from the intervertebral disc and vertebral bodies (21). Given that most gunshot wounds do not impair spinal stability, stabilizing aids are usually not required. Hence, such cases require minimally invasive surgical treatments.

There are some published reports on the microsurgical removal of a wounding body through a tube retractor through a posterior access along the optimal trajectory. This method proved to be very efficient in terms of regression of neurological dysfunctions and prevention of infectious complications in a spine gunshot wound (10, 22).

Nowadays, the least invasive surgical method in spinal surgery is percutaneous video endoscopy. Advantages of this method, like any minimally invasive technology, are well known and relate to clinical and economic aspects. Technical characteristics of spinal percutaneous endoscopic interventions ensure a targeted approach to a surgical target both through natural anatomical spaces of the vertebral segments (interlaminar space, intervertebral foramen), and through intervertebral discs and bone structures (23). Such interventions greatly reduce infectious complications afterwards (24). Although there are lots of papers devoted to full-endoscopic spinal surgery, its use in spine gunshot wounds has not yet been thoroughly discussed (2).

Of course, percutaneous video endoscopic aids are still inferior to open access in terms of comprehensive revision, sanitation and drainage of gunshot wounds, especially in case of multiple spinal injuries (shot, buckshot, etc.) (10). Disadvantages of full-endoscopic removal of a bullet from the spinal canal include the impossibility of complete sealing of defects in the dura mater. However, puncture endoscopic access, apparently, prevents appearance of cerebrospinal fluid cysts and fistulas. Clinical application of percutaneous unilateral biportal endoscopic technique (25, 26) and technical aids for full-endoscopic closure of defects in the dura mater of the spinal cord (27) promise a successful solution of such problems in future.

## Conclusion

Full-endoscopic operations can be effective in bullet wounds to the spine. Their application allows:

1) to remove the bullet from the spinal canal or intervertebral disc;
2) to inspect the epidural and subdural spaces of the spine;
3) reduce the risk of infectious complications through minimal invasiveness, sanitation and continuous intraoperative irrigation of the gunshot wound with saline sodium chloride solution;
4) perform plasty of the dura mater defect.

These operations should be performed at the tertiary care level by surgeons with sufficient experience in percutaneous endoscopic spinal surgery.

## Data Availability Statement

The original contributions presented in the study are included in the article/Supplementary Material, further inquiries can be directed to the corresponding author/s.

## Ethics Statement

Ethical review and approval was not required for the study on human participants in accordance with the local legislation and institutional requirements. The patients/participants provided their written informed consent to participate in this study. Written informed consent was obtained from the individual(s) for the publication of any potentially identifiable images or data included in this article.

## Author Contributions

MK, GB, and SM contributed to the conception and design of the study, the analysis and interpretation of data, and the work draft. MK designed figures and video. VM and VB offered guidance in study design and revised the article critically for important intellectual content. All authors read and approved the final version of the manuscript.

## Conflict of Interest

The authors declare that the research was conducted in the absence of any commercial or financial relationships that could be construed as a potential conflict of interest.

## Publisher's Note

All claims expressed in this article are solely those of the authors and do not necessarily represent those of their affiliated organizations, or those of the publisher, the editors and the reviewers. Any product that may be evaluated in this article, or claim that may be made by its manufacturer, is not guaranteed or endorsed by the publisher.

## Supplementary Material

Supplementary Video 1 Full-endoscopic surgery for gunshot wound of the lumbar spine: a report of case.

Supplementary Video 2 Full-endoscopic transforaminal surgery for gunshot wound of the lumbar spine: a report of case.

## References

[1] Shin JK Youn MS Seong YJ Goh TS Lee JS. Iatrogenic dural tear in endoscopic lumbar spinal surgery: full endoscopic dural suture repair (Youn's technique). Eur Spine J. (2018) 27:544–8. 10.1007/s00586-018-5637-6 29789920

[2] Telfeian AE Veeravagu A Oyelese AA Gokaslan ZL. A brief history of endoscopic spine surgery. Neurosurg Focus. (2016) 40:E2. 10.3171/2015.11.FOCUS15429 26828883

[3] Wagner R Telfeian AE Iprenburg M Krzok G Gokaslan Z Choi DB. Transforaminal endoscopic solution to a kyphoplasty complication. World Neurosurg. (2016) 91:195–8. 10.1016/j.wneu.2016.04.013 27072335

[4] Ito M Abumi K Kotani Y Kadoya K Minami A. Clinical outcome of posterolateral endoscopic surgery for pyogenic spondylodiscitis: results of 15 patients with serious comorbid conditions. Spine. (2007) 32:200–6. 10.1097/01.brs.0000251645.58076.96 17224815

[5] Sentürk S Ünsal ÜÜ. Percutaneous full-endoscopic removal of lumbar intradural extramedullary tumor via translaminar approach. World Neurosurg. (2019) 125:146–9. 10.1016/j.wneu.2019.01.206 30763742

[6] Konakondla S Sofoluke N Xia J Grant R Telfeian AE Hofstetter CP. Transforaminal endoscopic approach for large-sample tumor biopsy using beveled working channel for core technique: a technical note. World Neurosurg. (2020) 141:346–51. 10.1016/j.wneu.2020.05.096 32442734

[7] Schoenfeld AJ Laughlin MD McCriskin BJ Bader JO Waterman BR Belmont PJ. Spinal injuries in United States military personnel deployed to Iraq and Afghanistan: an epidemiological investigation involving 7877 combat casualties from 2005 to 2009. Spine. (2013) 38:1770–8. 10.1097/BRS.0b013e31829ef226 23759821

[8] Beaty N Slavin J Diaz C Zeleznick K Ibrahimi D Sansur CA. Cervical spine injury from gunshot wounds. J Neurosurg Spine. (2014) 21:442–9. 10.3171/2014.5.SPINE13522 24926931

[9] Jakoi A Iorio J Howell R Zampini JM. Gunshot injuries of the spine. Spine J. (2015) 15:2077–85. 10.1016/j.spinee.2015.06.007 26070284

[10] Crutcher CL Wilson JM DiGiorgio AM Fannin ES Shields JA Morrow KD. Minimally invasive Management of Civilian Gunshot Wounds to the lumbar spine: a case series and technical report. Operative Neurosurg. (2020) 19:219–25. 10.1093/ons/opaa030 32147736

[11] Farmer JC Vaccaro AR Balderston RA Albert TJ Cotler J. The changing nature of admissions to a spinal cord injury center: violence on the rise. J Spinal Disord. (1998) 11:400–3. 10.1097/00002517-199810000-00006 9811100

[12] Bono C Heary F. Gunshot wounds to the spine. Spine J. (2004) 4:230–40. 10.1016/S1529-9430(03)00178-5 15016402

[13] Mogila VV Maksimov SA. Features spine gunshot wounds and spinal cord in the lumbar – sacral. Tavricheskiy Mediko Biologicheskiy Vestnik. (2013) 16:123–5.

[14] Jaiswal M Mittal RS. Concept of gunshot wound spine. Asian Spine J. (2013) 7:359–64. 10.4184/asj.2013.7.4.359 24353856 PMC3863665

[15] Volkov PV Grin AA. Surgical treatment strategy at patients with gun and stab wounds of vertebrae and spinal cord. Russian J Neurosurg. (2010) 2:72–9.

[16] Sidhu GS Ghag A Prokuski V Vaccaro AR Radcliff KE. Civilian gunshot injuries of the spinal cord: a systematic review of the current literature spine. Clin Orthop Relat Res. (2013) 471:3945–55. 10.1007/s11999-013-2901-2 23479233 PMC3825909

[17] Bumpass DB Buchowski JM Park A Gray BL Agarwal R Baty J. An update on civilian spinal gunshot wounds. Spine. (2015) 40:450–61. 10.1097/BRS.0000000000000797 25811133

[18] Hakan T Çerçi A Gürcan S Akçay S. Firearm bullet settling into the lumbar spinal canal without causing neurological deficit: a report of two cases. Surg Neurol Int. (2016) 7:S251–4. 10.4103/2152-7806.181978 27213110 PMC4866057

[19] Çiftçi U Arinci AT Delen E Güçlühan D. Incomplete isolated C7 root injury caused by gunshot wound: a case report. Korean J Neurotrauma. (2017) 13:45–49. 10.13004/kjnt.2017.13.1.45 28512618 PMC5432449

[20] Apte A Bradford K Dente C Smith RN. Lead toxicity from retained bullet fragments: a systematic review and meta-analysis. J Trauma Acute Care Surg. (2019) 87:707–16. 10.1097/TA.0000000000002287 30939573

[21] Volkov PV Sorokin KV. Long terms results of non-penetrative gunshot wound of lumbar spine with prevertebral abscess forming. Russian J Neurosurg. (2011) 4:69–73.

[22] Shen FHT Samartzis D. Operative management of a sacral gunshot injury via minimally invasive techniques and instrumentation. Asian Spine J. (2013) 7:44–9. 10.4184/asj.2013.7.1.44 23508557 PMC3596584

[23] Hofstetter CP Ahn Y Choi G Gibson JNA Ruetten S Zhou Y. AOSpine consensus paper on nomenclature for Working-Channel endoscopic spinal procedures. Global Spine J. (2020) 10:111S−21S. 10.1177/2192568219887364 32528794 PMC7263337

[24] Liu X Yuan S Tian Y Wang L Gong L Zheng Y. Comparison of percutaneous endoscopic transforaminal discectomy, microendoscopic discectomy, and microdiscectomy for symptomatic lumbar disc herniation: minimum 2-year follow-up results. J Neurosurg Spine. (2018) 28:317–25. 10.3171/2017.6.SPINE172 29303471

[25] Heo DH Son SK Eum JH Park CK. Fully endoscopic lumbar interbody fusion using a percutaneous unilateral biportal endoscopic technique: technical note and preliminary clinical results. Neurosurg Focus. (2017) 43:E8. 10.3171/2017.5.FOCUS17146 28760038

[26] Kim JE Yoo HS Choi DJ Park EJ Jee SM. Comparison of minimal invasive versus biportal endoscopic transforaminal lumbar interbody fusion for single-level lumbar disease. Clin Spine Surg. (2020) 34:E64–71. 10.1097/BSD.0000000000001024 33633061 PMC8035997

[27] Park HJ Kim SK Lee SC Kim W Han S Kang SS. Dural tears in percutaneous biportal endoscopic spine surgery: anatomical location and management. World Neurosurg. (2020) 136:578–85. 10.1016/j.wneu.2020.01.080 31958589

